# Supplementary material for: Predicting visual function by interpreting a neuronal wiring diagram
Source: Nature. 2024 Oct 2;634(8032):113–23. doi: 10.1038/s41586-024-07953-5 (PMC11446822; doi:10.1038/s41586-024-07953-5)
Supplement: Supplementary file 6 — CRF and ERF predictions for individual TmY4 and TmY9 cells. Analogous to Supplementary Data 3, but for TmY target types. Shown are the top four monosynaptic pathways, the strongest pathway passing through each of the top ten intermediary types (ranking from Extended Data Fig. 7), and the trisynaptic pathway Tm1–TmY–Dm3–TmY (see the section entitled Prediction of spatial normalization). [file 41586_2024_7953_MOESM6_ESM.zip › DataS4/TmY9qΓèÑ/720575940611671250.pdf]

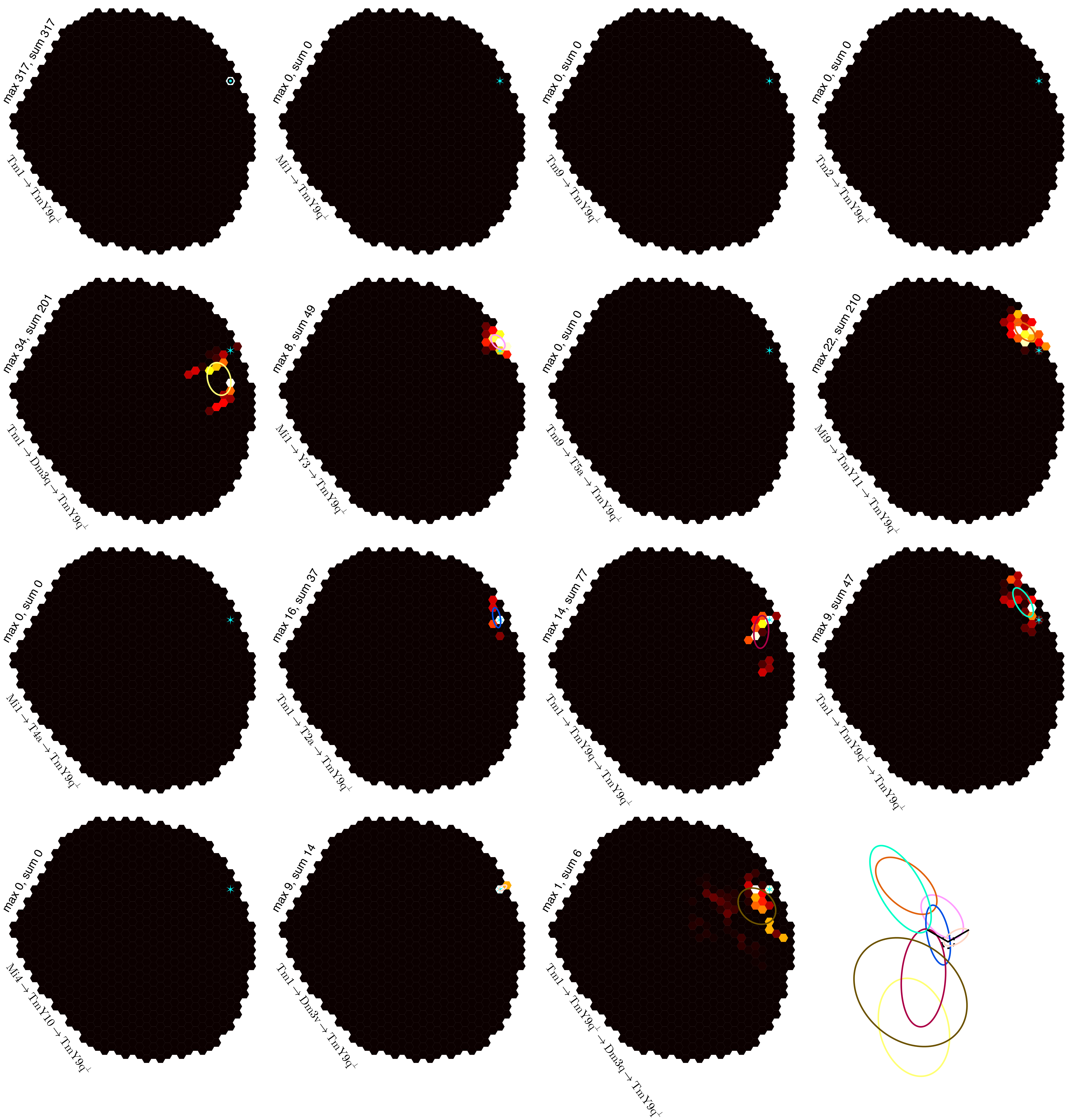
max 317, sum 317
Tm1 → TmY9q⊥
max 0, sum 0
Mi1 → TmY9q⊥
max 0, sum 0
Tm9 → TmY9q⊥
max 0, sum 0
Tm2 → TmY9q⊥
max 34, sum 201
Tm1 → Dm3q → TmY9q⊥
max 8, sum 49
Mi1 → Y3 → TmY9q⊥
max 0, sum 0
Tm9 → T5a → TmY9q⊥
max 22, sum 210
Mi9 → TmY11 → TmY9q⊥
max 0, sum 0
Mi1 → T4a → TmY9q⊥
max 16, sum 37
Tm1 → T2a → TmY9q⊥
max 14, sum 77
Tm1 → TmY9q → TmY9q⊥
max 9, sum 47
Tm1 → TmY9q⊥ → TmY9q⊥
max 0, sum 0
Mi4 → TmY10 → TmY9q⊥
max 9, sum 14
Tm1 → Dm3v → TmY9q⊥
max 1, sum 6
Tm1 → TmY9q⊥ → Dm3q → TmY9q⊥
